# Comparative profiling between primary colorectal carcinomas and metastases identifies heterogeneity on drug resistance

**DOI:** 10.18632/oncotarget.11570

**Published:** 2016-08-24

**Authors:** Feng Luo, Jinbang Li, Shigang Wu, Xuefang Wu, Meixiang Chen, Xueyun Zhong, Kunping Liu

**Affiliations:** ^1^ Department of Pathology, Qingyuan People's Hospital, Jinan University, Qingyuan 511518, China; ^2^ Department of Pathology, Medical College, Jinan University, Guangzhou 510632, China

**Keywords:** colorectal cancer, drug resistance, WNT signaling pathway, EMT, cancer stem cells

## Abstract

Metastases cause recurrence and mortality for patients with colorectal carcinomas (CRC). In present study, we evaluated heterogeneity on drug resistance and its underlying mechanism between metastatic and primary CRC. Immunohistochemical results from clinical tissue microarray (TMA) suggested that the expression concordance rates of cancer stem cells (CSCs) and drug resistance relative proteins between lymph-node metastatic and primary CRC foci were low. The apoptotic and proliferation indexes in metastasis CRC specimens were decreased compared with primary. In vitro experimental results indicated that the migration and invasion abilities were upregulated in metastatic cells SW620 compared with primary cells SW480, the cellular efflux ability and WNT/β-catenin activity were also upregulated in SW620 cells. After 5-fluorouracil (5-Fu) treatment, the reduction in the proportion of cell apoptosis, CD133 and TERT expression levels in SW620 were lower than that in SW480 cells. Bioinformatics analysis in whole-genome transcriptional profiling results between metastatic and primary CRC cells suggested that differentially expressed genes were mainly centered on well-characterized signaling pathways including WNT/β-catenin, cell cycle and cell junction. Collectively, heterogeneity of drug resistant was present between metastatic and primary CRC specimens and cell lines, the abnormal activation of WNT/β-catenin signaling pathway could be a potential molecular leading to drug resistant ability enhancing in metastatic CRC cells.

## INTRODUCTION

Colorectal cancer (CRC) is one of the most common malignancies in the world. Approximately 25% to 50% of colorectal cancer patients develop metastatic disease [1]. Once metastasis has occurred in CRC, a complete cure of the disease is unlikely. Colorectal liver metastasis (CLM), occurring in about 20% of CRC patients during the course of their treatment, is the most common distant metastasis from CRC [2, 3].

The phenotype of human CRC cells mainly depends on the interaction of genetic and environmental factors. Numerous researches have pointed out that the heterogeneity was formed during and after the process of epithelial-mesenchymal transition (EMT) in CRC cells [3–5]. Thiruvengadam and colleagues reported that Zeb-1 and other regulators of EMT maintain drug resistance in human pancreatic cancer cells [6]. Moreover, the process of EMT leads to great increases in the number of self-renewing cells that can initiate the seeding of mammospheres, which raises the possibility for achieving EMT process, at the same time, may also impart self-renewal and multidrug resistance capabilities to metastatic cancer cells [7].

Chemotherapy has synergistic effects with radiotherapy and surgery and play vital roles in combined therapy for CRC [8, 9]. Chemotherapy insensitivity and poor prognosis are biological characteristics of metastases CRC [10], but little is known about the biological behaviors differences between primary and metastatic CRC, especially at difference of drug resistance. Therefore, there is a need for better understanding of the molecular mechanisms underlying the metastatic phenotype that may provide information leading to the development of drugs to control or prevent metastatic disease.

In present study, to a much better understanding of biological differences between primary and metastatic CRC, we used high-throughput DNA microarrays to compare differentially expressed genes of primary and metastatic CRC cells. The migration and invasion abilities difference between primary CRC cells SW480 and metastatic cells SW620 were determined through transwell and boyden chamber assays. We found that the ability of drug resistant in metastatic cells SW620 was greater than primary colorectal cancer cells SW480 owing to cancer stem cells and drug resistance relative proteins activation, which partially reconfirmed by the clinical TMA IHC assay.

## RESULTS

### Comparison of the CSCs and drug resistance relative proteins between primary CRC and colorectal lymphatic metastasis specimens

**Figure 1:**
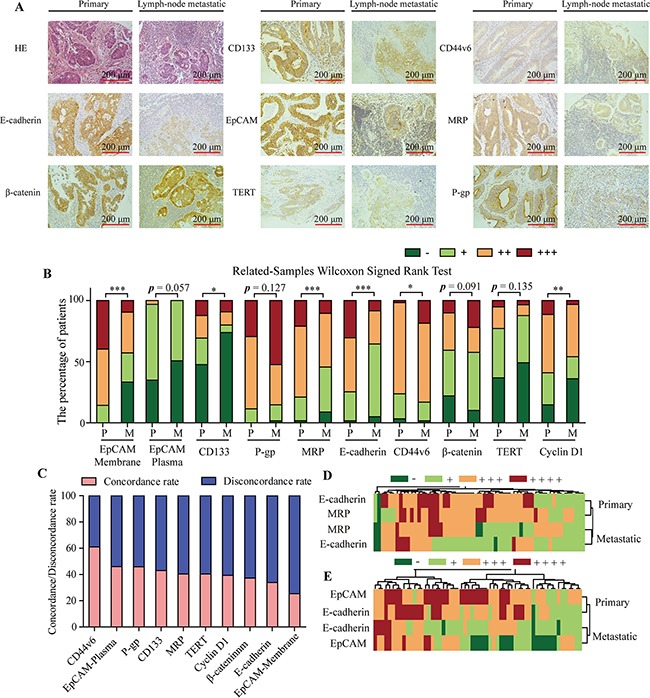
IHC staining assay on primary CRC and colorectal lymphatic metastasis specimens **A.** Representative IHC of CRC samples, showing the expression level of EpCAM, CD133, P-gp, MRP, E-cadherin, CD44v6, β-catenin and TERT between primary CRC and colorectal lymphatic metastasis groups. **B.** The percentage of patients with the negative (−), weak (+), moderate (++) and high (+++) expression of CSCs and drug resistance relative proteins in primary CRC and colorectal lymphatic metastasis groups. P, primary. M, lymphatic metastasis. **C.** The concordance/disconcordance rate of CSCs and drug resistance relative proteins between primary CRC and colorectal lymphatic metastasis groups. **D.** Hierarchical clustering of the E-cadherin and MRP protein expression levels was generated by R programming language based on IHC results in primary CRC and colorectal lymphatic metastasis groups. **E.** Hierarchical clustering of the E-cadherin and EpCAM protein expression levels was generated by R programming language based on IHC results in primary CRC and colorectal lymphatic metastasis groups. High and low expressed proteins are shown by red and green, respectively. Each bar represents the means ± SD. *p < 0.05, **p < 0.01, ***p < 0.001.

To gain insight into the expression level of CSCs and drug resistance relative proteins in CRC, we used the clinical tissue microarray to assess these protein expression levels between primary CRC and colorectal lymphatic metastasis specimens. The expression level of EpCAM (Membrane), E-cadherin, MRP, CD133 and Cyclin D1 were significantly upregulated in primary CRC foci compared with the corresponding lymph-node metastatic foci, whereas CD44v6 was downregulated in primary CRC foci (Figure 1A and 1B, *p* < 0.05). In addition, although there was no significant difference in the expression level of β-catenin between primary CRC and colorectal lymphatic metastasis specimens overall, the ratio of high expression (+++) of β-catenin was signifcantly higher in lymph-node metastatic foci (22.03%) compared with the corresponding primary CRC foci (10.16%, Figure 1A and 1B).

The concordance rates of the CSCs and drug resistance relative proteins between the primary CRC foci and corresponding lymph-node metastatic foci were showed in Figure 1C. The concordance rates were low, and there was only one protein, CD44v6, with over 50% concordance rate. To a better understanding the relationships of these CSCs and drug resistance relative proteins between the primary CRC foci and corresponding lymph-node metastatic foci, hierarchical clustering was generated by R programming language based on IHC results. Statistical analysis revealed that the expression level of E-cadherin was positively correlated with MRP and EpCAM expression in both primary CRC foci and corresponding lymph-node metastatic foci (Figure 1D and 1E, Table 1 and 2). Meanwhile, the expression level of EpCAM (Membrane), E-cadherin and MRP were significantly higher in primary CRC foci compared with the corresponding lymph-node metastatic foci (Table 1 and 2, *p* < 0.001).

**Table 1:** Immunohistochemical analysis of E-cadherin and MRP in CRC in vivo

	Primary-E-cadherin	Metastatic-MRP
Primary-MRP	*p* < 0.01, *r* = 0.353^a^	*p* < 0.001^b^
Metastatic-E-cadherin	*p* < 0.001^b^	*p* < 0.05, *r* = 0.278^a^

a Spearman's correlation Test,

b Related-Samples Wilcoxon Signed Rank Test

**Table 2:** Immunohistochemical analysis of E-cadherin and EpCAM in CRC in vivo

	Primary-EpCAM	Metastatic-E-cadherin
Primary-E-cadherin	*p* < 0.05, *r* = 0.269^a^	*p* < 0.001^b^
Metastatic-EpCAM	*p* < 0.001^b^	*p* < 0.05, *r* = 0.300^a^

a Spearman's correlation Test

b Related-Samples Wilcoxon Signed Rank Test

### Comparison of the proliferation and apoptotic index between primary CRC and colorectal lymphatic metastasis specimens

**Figure 2:**
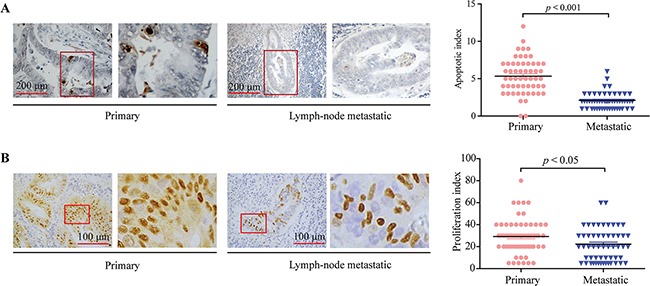
TUNEL and Cyclin D1staining assays on primary CRC and colorectal lymphatic metastasis specimens **A.** Left, TUNEL staining assay on primary CRC and colorectal lymphatic metastasis specimens. Right, the apoptotic index in primary and metastatic groups. **B.** Left, Cyclin D1 staining assay on primary CRC and colorectal lymphatic metastasis specimens. Right, the proliferation index in primary and metastatic groups. Each bar represents the means ± SD. *p < 0.05, **p < 0.01, ***p < 0.001.

The apoptosis of CRC specimens was examined using TUNEL staining assay in CRC specimens, the apoptotic index of colorectal lymphatic metastasis specimens was significantly decreased compared to primary CRC specimens (Figure 2A, *p* < 0.001). The proliferation index of CRC specimens was also examined using Cyclin D1 staining assay in CRC specimens (Figure 2B), we found that the proliferation index of colorectal lymphatic metastasis specimens was significantly decreased compared to primary CRC specimens (*p* < 0.05).

### The adhesive activity was downregulated, the efflux rate of Rh123 and WNT/β-catenin activity were upregulated in metastatic cells SW620

**Figure 3:**
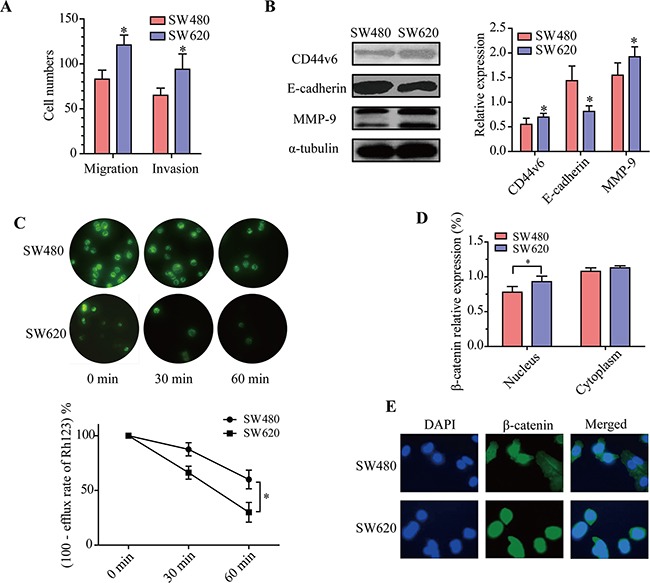
Comparison of adhesive activity, P-gp function and WNT/β-catenin activity between SW480 and SW620 cells **A.** Transwell assays and boyden chamber assays evaluated the migration and invasion abilities of SW480 and SW620 cells. **B.** Western blot analyzed of the expression levels of CD44v6, MMP-9 and E-cadherin in SW480 and SW620 cells. Each bar represents the means ± SD. **C.** The fluorescent signals of Rh123 before and after 5-Fu treatment were observed by fluorescent microscope (200X) in SW480 and SW620 cells. **D.** Western blot analysis the expression level of β-catenin protein in cytoplasmic and nuclear of SW480 and SW620 cells. Values are expressed as protein/α-tubulin or protein/Histon. The density of the protein band was quantitated using Quantity One software. The data are expressed as mean ± SD of three experiments. * *p* < 0.05 compared with the SW480 cells. **E.** Immunofluorescence (400X) was done to visualize the expression and nuclear accumulation of β-catenin between SW480 and SW620 cells.

The migration and invasion abilities difference between SW480 and SW620 cells were determined through transwell assays and boyden chamber assays. As shown in Figure 3A, the migration and invasion abilities of SW620 cells were significantly greater than SW480 cells (*p* < 0.05). Detection of the key components of cell migration and invasion pathways by western blotting demonstrated that compared to SW480 cells, the expression levels of CD44v6 and MMP-9 were elevated, while the level of E-cadherin was decreased in SW620 cells (Figure 3B, *p* < 0.05). These results demonstrated that metastatic cells SW620 have greater migration and invasion abilities than primary colorectal cancer cells SW480.

To examine the P-gp function between SW480 and SW620 cells, the fluorescence intensity of Rh123 was measured by flow cytometry and the efflux rate of Rh123 was calculated. As shown in Figure 3C, the efflux rate of Rh123 was higher in SW620 cells than that in SW480 cells (*p* < 0.05). More interestingly, western blot analysis of β-catenin proteins in cytoplasmic and nuclear displayed that β-catenin was upregulated in the nuclei of SW620 compared to SW480 cells (Figure 3D, *p* < 0.05). Immunofluorescence analysis verified that β-catenin was more favored distributed in the nuclei of SW620 cells compared with SW480 cells (Figure 3E).

### Cancer stem cells and drug resistance relative proteins activation lead to metastatic cells SW620 drug resistant ability enhancing

**Figure 4:**
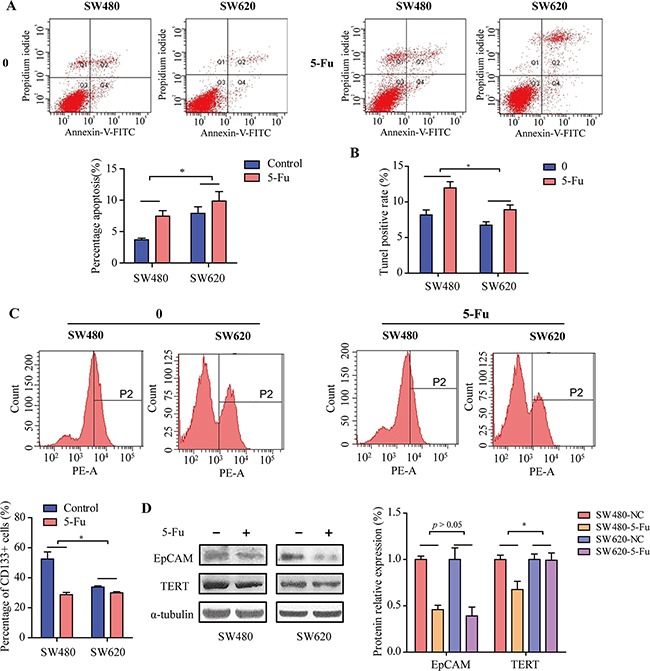
The effects of 5-Fu on cancer stem cells and drug resistance relative proteins of SW480 and SW620 cells **A.** The rate of Annexin V positive cells before and after 5-Fu treatment in SW480 and SW620 cells. All values are presented as mean±SD of three experiments. **B.** CRC cells cultured in the presence of 10% serum on coverslips were kept in the absence and presence of 50 μmol/L 5-Fu for 3 days, at which point the cells were fixed and processed for TUNEL staining, to detect cells under-going programmed cells death. **C.** Flow cytometric analyzed the percentage of CD133+ cells when treated with 5-Fu or control in SW480 and SW620 cells. **D.** Expression levels of TERT and EpCAM in SW480 and SW620 cells before and after treatment with 5-Fu for 24h. * *p* < 0.05.

After incubated with 5-Fu, the apoptosis induced by 5-Fu of SW480 and SW620 cells was examined using flow cytometry after Annexin V-FITC/PI staining (Figure 4A). We found that 5-Fu could significantly increase the apoptosis in SW480 compared to SW620 cells (Figure 4A, *p* < 0.05). The apoptosis induced by 5-Fu of CRC cells was also examined using TUNEL staining assay. Similarly, we found that 5-Fu could significantly increase the apoptosis in SW480 compared to SW620 cells (Figure 4B, *p* < 0.05). These results suggested that metastatic cells SW620 have greater drug resistant ability than primary colorectal cancer cells SW480.

To investigate the underlying mechanisms leading to SW620 drug resistant ability enhancing, we assessed whether these effects of SW620 cells was due to cancer stem cells and drug resistance relative proteins activation. After either treated with 5-Fu or control for 24 h, the effect of 5-Fu on the proportion of CD133+ of SW480 and SW620 cells was examined using flow cytometry. As shown in Figure 4C, the proportions of CD133+ SW480 cells were significantly decreased compared to SW620 cells (*p* < 0.05). As shown in Figure 4D, following 24 h treatment of 5-Fu, the expression level of TERT was downregulated in SW480 cells, whereas these effects were slight in SW620 cells. In addition, the expression levels of EpCAM were downregulated following 24 h treatment of 5-Fu in both SW480 and SW620 cells (Figure 4D, *p* < 0.05).

### Screening differentially expressed genes based on microarray data between metastatic and primary CRC

In order to survey the differentially expressed genes between metastatic and primary CRC, we download cDNA microarrays from GEO database (No. GSE22834) and performed supervised analysis using SAM and identified 1,067 unique genes with aberrant expression in liver metastatic CRC compared to primary CRC. Functional annotations of the differentially expressed genes were generated using the GenCLiP 2.0 online tool.

**Figure 5:**
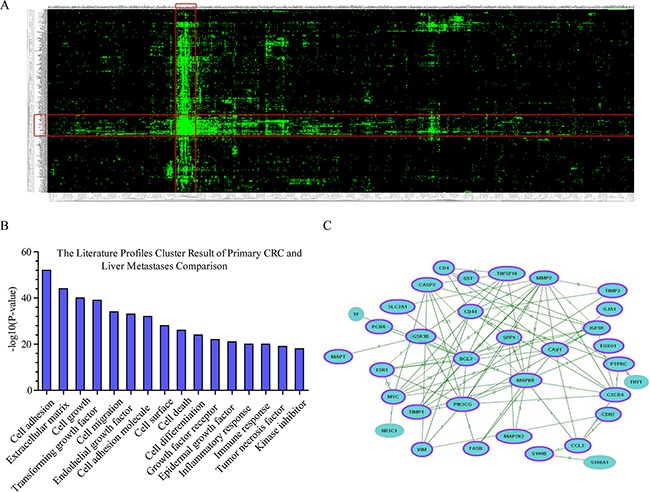
Functional annotations of the differentially expressed genes based on GenCLiP 2.0 online tool analyzed between CLM and primary CRC **A.** Heatmap showed the clustering result generated from differentially expressed genes and literature profiles-based keywords based on GenCLiP 2.0 online tool analysis between CLM and primary CRC. Each row and column in the heat map representation is literature profiles-based keywords and differentially expressed genes, respectively. **B.** The literature profiles-based keywords cluster result of primary CRC and liver metastases comparison. **C.** Gene-gene interaction network of primary CRC and liver metastases comparison generated using the GenCLiP 2.0 online tool. The purple band mean genes in the network related with CRC according to the GenClip 2.0 literature data mining results. Related genes shown with purple band.

As shown in Figure 5A, heatmap showing the differentially expressed genes and literature profiles-based keywords based on GenCLiP 2.0 online tool analyzed between metastatic and primary CRC. With the results of clustering, the 16 highest-ranking literature profiles-based keywords of primary CRC and liver metastases comparison were listed in Figure 5B. We screened the most relevant genes associated with these keywords. Gene-gene interaction network of these 34 genes were generated and showed in Figure 5C and Table 3 using the GenCLiP 2.0 online tool.

**Table 3:** The key differentially expressed genes between CLM and primary CRC

Gene	Co-genes	Gene name
BCL2	14	B-cell CLL/lymphoma 2
MAPK8	13	Mitogen-activated protein kinase 8
MMP2	12	Matrix metallopeptidase 2
PIK3CG	12	Phosphatidylinositol-4,5-bisphosphate 3-kinase catalytic subunit gamma
CD44	8	CD44 molecule (Indian blood group)
CXCR4	8	Chemokine (C-X-C motif) receptor 4
IGF1R	8	Insulin like growth factor 1 receptor
MYC	8	V-myc avian myelocytomatosis viral oncogene homolog
CASP3	7	Caspase 3
CAV1	7	Caveolin 1
ESR1	6	Estrogen receptor 1
SPP1	6	Secreted phosphoprotein 1
TIMP1	6	TIMP metallopeptidase inhibitor 1
TNFSF10	6	Tumor necrosis factor superfamily member 10
CCL2	5	Chemokine (C-C motif) ligand 2
GSK3B	5	Glycogen synthase kinase 3 beta

Gene names are according to the National Center for Biotechnology Information. Co-genes: Co-occurrence of gene pair and keyword(s) in Literature Mining Gene Networks.

### Screening differentially expressed genes based on microarray data between SW480 and SW620 cells

Next, we extracted total RNA and conducted cDNA microarrays analysis (Roche, NimbleGen) between CRC cells derived from human primary colorectal cancer cells SW480 and their metastatic cells SW620. We then performed supervised analysis using SAM and identified 1,362 unique genes with aberrant expression in SW620 when compared to SW480 cells. Functional annotations of the differentially expressed genes were generated using the GenCLiP 2.0 online tool.

**Figure 6:**
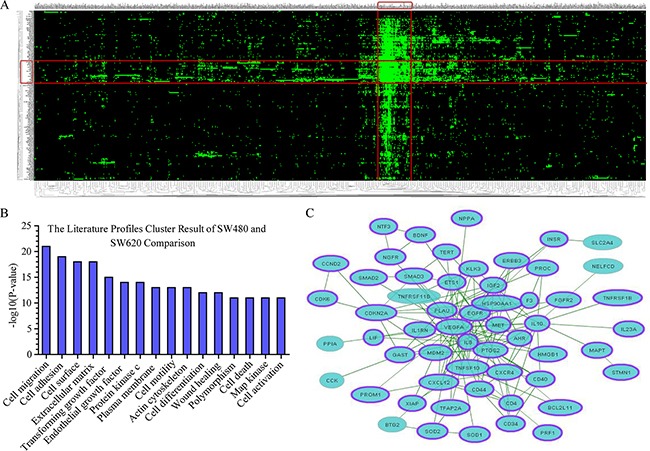
Functional annotations of the differentially expressed genes based on GenCLiP 2.0 online tool analyzed **A.** Heatmap showed the clustering result generated from differentially expressed genes and literature profiles-based keywords based on GenCLiP 2.0 online tool analysis between SW480 and SW620 cells. Each row and column in the heat map representation is literature profiles-based keywords and differentially expressed genes, respectively. **B.** The literature profiles-based keywords cluster result of SW480 and SW620 comparison. **C.** Gene-gene interaction network of SW480 and SW620 comparison generated using the GenCLiP 2.0 online tool. The purple band mean genes in the network related with CRC according to the GenClip 2.0 literature data mining results. Related genes shown with purple band.

As shown in Figure 6A, heatmap showing the differentially expressed genes and literature profiles-based keywords based on GenCLiP 2.0 online tool analyzed between SW480 and SW620 cells. With the results of clustering, the 16 highest-ranking literature profiles-based keywords of SW480 and SW620 cells comparison were listed in Figure 6B. We screened the most relevant genes associated with these keywords. Gene-gene interaction network of these 56 genes were generated and showed in Figure 6C using the GenCLiP 2.0 online tool.

### Comparison of the differentially expressed genes between CRC primary-metastasis tissues and SW480-SW620 cell lines

**Figure 7:**
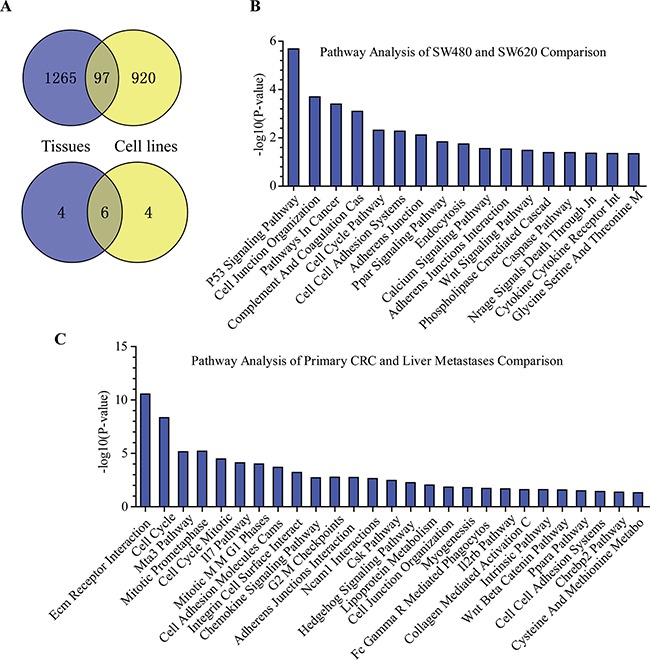
Comparison results of the differentially expressed genes between CRC primary-metastasis tissues and SW480-SW620 cell lines **A.** Overlapping and nonoverlapping genes and keywords between CRC primary-metastasis tissues and SW480-SW620 cell lines. **B.** Pathway analysis of SW480 and SW620 comparison. **C.** Pathway analysis of primary CRC and liver metastases comparison.

As noted above, microarray screening analysis found that a total number of 1,067 genes were differentially expressed between the metastatic and primary CRC, while 1,362 genes were differentially expressed between SW480 and SW620 cells. As shown in Figure 7A, the overlapping differentially expressed genes between the CRC tissues and cell lines were 97 genes; however, the overlapping among the top 10 highest-ranking keywords between the CRC tissues and cell lines were 6 (Figure 7A).

The differentially signaling pathways were analyzed using the GenCLiP 2.0 online tool. As shown in Figure 7C, the histogram showing the differentially signaling pathways between metastatic and primary CRC. The various aberrant signaling pathways between metastatic and primary CRC indicated that heterogeneity was present among different tumors as well as between primary and metastatic CRC foci. The differentially signaling pathways between SW480 and SW620 cells were also showed in Figure 7B.

## DISCUSSION

Metastases cause significant recurrence and mortality for patients with CRC, and the survival rates with these case remain unacceptably low [1, 2, 10]. The process of EMT often considered being the switch that allows tumor cells to acquire the capacity to invade and ultimately metastasize to distant sites [11, 12]. Numerous researches have pointed out that the genetic heterogeneity was present among different tumors as well as between primary and metastatic cancer cells [13, 14]. However, the biological differences between primary and metastatic CRC, especially at difference of drug resistance is still largely unknown. Therefore, elucidation of molecular mechanism underlying the metastatic phenotype is critical for the development of potential therapeutic agents for CRC.

DNA microarrays is a recent development and application of human genome and high-throughput technology, allow us to simultaneously examine thousands of genes, provided a promising way to much better understanding of CRC carcinogenesis [15–17]. The overlapping differentially expressed genes between the CRC tissues and cell lines were rare, but the overlap ratio among these top 10 highest-ranking keywords was 60%. These results suggested that although differentially expressed genes are dissimilarity, the functions of these genes are mainly similar between CRC tissues and cell lines. To compare the biological behavior difference between primary and metastatic CRC cells, the migration and invasion abilities difference between SW480 and SW620 cells were determined through transwell and boyden chamber assays. Our findings reveal that metastatic CRC SW620 cells have greater migration and invasion abilities than primary SW480 cells, the relative proteins CD44v6 and MMP-9 were upregulated, E-cadherin was downregulated in SW620 cells. These results consistent with Palmieri and Kubens' experimental results [18, 19].

In present study, the immunohistochemistry results showed that the expression level of drug resistance and stem cell related proteins in metastatic and primary CRC foci were different. The concordance rates of these proteins between the lymphatic metastatic CRC foci and primary foci were low, and there was only one protein, CD44v6, with over 50% concordance rate. In addition, the apoptotic and the proliferation index in lymphatic metastasis CRC foci were decreased compared with primary foci. These results showed that the heterogeneity at difference expression level of drug resistance and stem cell related proteins was present between the metastatic and primary CRC foci, suggested that biological behaviors between metastatic and primary cells may be different. In vitro, we found that the efflux rate of Rh123 was upregulated in metastasis CRC SW620 cells than primary SW480 cells. According to the flow cytometry and TUNEL staining experimental results, we found that reduction in the proportion of apoptosis in SW620 cells induced by 5-Fu treatment were lower than that in SW480 cells. After treated with 5-Fu, reduction in the proportion of CD133 and TERT protein expressions in SW620 cells were lower than SW480 cells. These results indicated that metastatic cells with such characteristics are partly responsible for its drug resistant ability enhancing. Perhaps this explains in part why the lower remission and higher recurrent were happened to patients with metastasis after chemotherapy treatment.

Analysis results from GenCLiP 2.0 suggested that WNT pathway was involved in aberrant signaling pathways between metastatic and primary CRC, as well as between SW480 and SW620 cells. The main oncoprotein in colorectal cancer is the WNT pathway effector β-catenin, which transportation to the nucleus and overactivation due to genic mutations in the APC, Axin, CKI, and GSK-3β in most cases [20–25]. Aberrant WNT signaling pathway is associated with a wide array of tumor types and plays an important role in the maintenance of stemness of CSCs and drug resistant ability [26–31]. Our experimental results show that β-catenin was upregulated and accumulated in the nuclei, whereas E-cadherin was downregulated in SW620 compared with SW480 cells. Typically, E-cadherin and β-catenin located at the cell membranes and constituted intercellular junctions [32–36]. During the process of EMT, the E-cadherin/β-catenin complex breakdown, accompanying with β-catenin translocation into nuclei and WNT signaling pathway over activation in CRC [37–41]. In addition, our previous research shows that the WNT signaling pathway plays an important role in drug resistance in colon cancer cells by alternating the expression level of CSC markers and drug resistance relative proteins [42, 43]. Junlin and colleagues also reported that the activation of β-catenin and Akt pathways by Twist are critical for the maintenance of EMT associated cancer stem cell-like characters [44].

In conclusion, we show for the first time that the heterogeneity on drug resistance was present between metastatic CRC foci and primary foci and cell lines, suggested that the molecular pathological diagnosis and follow-up clinical therapies for primary CRC foci may not be justly suitable for the corresponding metastatic foci. WNT/β-catenin signaling pathway could be a potential molecular leading to drug resistant ability enhancing in metastatic CRC cells.

## MATERIALS AND METHODS

### Datasets

The raw experimental data under accession no. GSE22834 used in the present study, contributed from Albert Y *et al* [45], is publically available in the Gene Expression Omnibus (GEO) database (http://www.ncbi.nlm.nih.gov/geo). These data, which include 31 primary CRC specimens from 30 patients (14 males and 16 females; age range: 36 to 80; stage I/II/III/IV=1/10/11/8, diagnosed between 2000 and 2004), and 32 CLM specimens from 31 patients (16 males and 15 females; age range: 40 to 82), were produced by cDNA microarrays. In the present study, using primary CRC specimens as control, the molecular variations in CLM specimens were identified by the GenCLiP 2.0 online tool [46] (http://ci.smu.edu.cn/).

### Patients and tissue samples

Paraffin-embedded surgical specimens of human primary CRC specimens and corresponding lymph-node metastatic specimens (from Department of Pathology, Qingyuan People's Hospital, Jinan University) were used for clinical tissue microarray expression profiling analysis. We used 60 primary CRC specimens and corresponding lymph-node metastatic specimens from 60 patients (36 males and 24 females; age range: 30 to 85; Duke stage III/IV=43/17, diagnosed between 2005 and 2008). For the use of these clinical materials for research purposes, prior written informed consent and ethics approval were obtained from all participants and the Ethics Committees of the Qingyuan People's Hospital, respectively.

### Cell culture and chemicals

Human colon adenocarcinoma SW480 and SW620 cells were obtained from American Type Culture Collection. The cells were cultured in RPMI 1640 medium (Gibco, USA) with 10% fetal bovine serum (TBD, China) at 37°C under an atmosphere of 5% CO_2_. 5-Fu was purchased from Shanghai Xudong Haipu Pharmaceutical Co., Ltd., China.

### Apoptosis analysis

For the apoptosis analysis, the cells were treated for 24 h with control or 5-Fu (50μmol/L). The cells were measured using FACSAriaflow cytometer (BD Biosciences, USA), and Annexin V (+) cells were counted for apoptotic cells after Annexin V-fluorescein isothiocyanate/propidium iodide (FITC/PI)(BD Pharmingen, USA) double staining. The experiment was performed in triplicate.

### Immunofluorescence and confocal microscopy

Cells were seeded onto glass cover-slips in 6-well plates at a density of 2×10^5^/well and incubated for 48 h. The cells were fixed in acetone at 20°C for 30 min. Then the cells were exposed to goat serum for 1 h and incubated with mouse anti-β-catenin (1:100) for 12 h at 4°C and FITC-labelled rabbit-anti-mouse IgG (1:300, Dingguo Biotechnology, China) for 1 h at 37°C. The cells were counterstained with 4′, 6-diamidino-2-phenylindole (DAPI) (Dingguo Biotechnology, China) for 10 min and analyzed using a laser scanning confocal microscope.

### Flow cytometry analysis of CD133 positive cell population

Human colon adenocarcinoma SW480 and SW620 cell lines (1×10^6^) were detached by treatment with 0.25% trypsin/EDTA and washed twice with phosphate-buffered saline. The cells were then resuspended in 100 μl of Staining Buffer containing 1% fetal bovine serum and place on ice for 20 min to block Fc receptors. After incubating with primary phycoerythrin antihuman CD133 antibody (Milteny, Germany) for another 10 min on ice in the dark, the cells were washed twice with 1 ml of ice-cold Staining Buffer and centrifuged (300×g) for 10 min at 4°C. Cells resuspended in 0.3 ml of 2% formaldehyde fixation buffer were analyzed using a FACSAriaflow cytometer and Cell Quest software (BD Biosciences). All flow cytometry results were obtained from two independent experiments performed in triplicate.

### Western blot analysis

Following treatment with different drugs for 24 h, the cells were collected and lysed. Protein content was measured by the BCA protein assay kit (Beyotime Institute of Biotechnology, Shanghai, China) and 20 μg protein per lane was separated by 8%-12% sodium dodecyl sulfate polyacrylamide gel electrophoresis (SDS-PAGE) and transferred onto nitrocellulose membranes. Specific protein bands were achieved with an ECL detection reagent (Pierce, Rockford, IL, USA). Anti-CD44v6 (Cell Signaling Technology, Danvers, MA, USA) dilution was 1:1000. Anti-β-catenin (Cell Signaling Technology) dilutions were 1:1,000. Anti-MMP-9 and anti-TERT (Abcam, Cambridge, MA, USA) dilutions were1:500 and 1:800. Anti-EpCAM and anti-α-tubulin (Cell Signaling Technology) dilutions were 1:500 and 1:1,000. Horseradish peroxidase (HRP)-conjugated goat-anti-rabbit and goat-anti-mouse IgG antibodies (ProteinTech Group, Chicago, IL, USA) dilutions were 1:3,000. α-tubulin was used as a protein loading control. The images were captured with ChemiDocTM CRS+ Molecular Imager (Bio-Rad, Hercules, CA, USA). The density of the protein band was quantitated using Quantity One software (Bio-Rad). The experiment was performed in triplicate.

### Rh123 efflux assay for P-gp function

After exposed to 5-Fu for 24 h, the CRC cells were incubated with 4 μg/ml rhodamine 123 (Rh123) (KeyGEN Biotech. Co., Ltd, China) for 30 or 60 min at 37°C. The fluorescence intensity of Rh123 in cells was detected by flow cytometry.

### Characterization of apoptosis morphology in cells

Cells were plated in 96-well plates at 3,000 cells per well. After compound treatment, cells were fixed using 10% buffered formalin/4% formaldehyde. Cellular DNA fragmentation morphology was detected by terminal deoxynucleotidyl transferase (TdT)-mediated nick end labeling (TUNEL) staining using ApopTag red in situ kit (Roche, AG., Germany) according to the manufacturer's directions. TUNEL-positive cells were visualized and analyzed using Cellomic ArrayScan II image analysis system (Cellomics, Inc., Pittsburgh, PA).

### Transwell migration and boyden chamber invasion assays

For the transwell migration assay, 10^5^ cells in 100 ml of serum-free DMEM media were triplicate seeded in each fibronectin-coated polycarbonate membrane insert in a transwell apparatus (Corning). 600 μl of 10% NCS in DMEM was added to the bottom chamber. SW480 and SW620 cells were incubated at 37°C for 12 h. The inserts were washed twice with prewarmed PBS. Cells adhered on the lower surface were fixed with 100% methanol at RT for 15min and stained with hematoxylin for 15min. Cell numbers in six predetermined fields in each replicate were counted under the microscope (Nikon ECLPSE 80i system; ×200). All assays were independently repeated at least for three times. Cell invasion assays were performed as the migration assay except the transwell membrane was precoated with 24mgml^−1^ Matrigel (R&D Systems) and the cells were incubated for 24 and 18 h, respectively.

### TMA construction and immunohistochemistry

For immunohistochemical staining (IHC), Anti-CD133 (ZSBIO, Beijing, China) dilution was 1:100. Anti-CD44v6 (ZSBIO) dilution was 1:200. Anti-Cyclin D1 (ZSBIO) dilutions were 1:50. Anti-MRP (ZSBIO) dilutions were 1:50. Anti-P-gp (ZSBIO) dilutions were 1:50. Anti-TERT (Abcam, Cambridge, MA, USA) dilutions was 1:200. Anti-β-catenin (Fuzhou Maixin Biotech. Co., Ltd., Fujian, China) dilutions were 1:1. Anti-E-cadherin (ZSBIO) dilutions was 1:70. Anti-EpCAM (Cell Signaling Technology, Danvers, MA, USA) dilutions was 1:800, and incubated overnight at 4°C. Chromogenic detection was then done using a peroxidase-conjugated secondary antibody and DAB reagents (ZSBIO). The proliferation index was analyzed using the Cyclin D1 antibody. Staining was measured as the percentage of positively stained nuclei in 200 tumor cells in a consecutive field. Counting was conducted on the most intensely stained areas. The immunostains were scored by two pathologists (FL and SGW) blinded to the clinical data.

### Statistical analysis

Statistical analysis was performed with the SPSS13.0 software package (SPSS Inc., Chicago, IL, USA). Two-class Significance Analysis of Microarrays (SAM) was used to identify genes that were differentially expressed in 31 CRC specimens and 32 CLM specimens, as well as metastatic cells SW620 and primary CRC cells SW480, the statistical significance was assessed by a false discovery rate (FDR). Data are presented as mean ± SD. One way analysis of variance (ANOVA) was used for apoptosis, cell cycle and western blot data analyses. Comparison of the CSCs and drug resistance relative proteins between primary CRC and colorectal lymphatic metastasis specimens was performed with the related-samples Wilcoxon signed rank test and Spearman's correlation test. *p* < 0.05 was considered to indicate a statistically significant difference.
